# Determination of high sensitive cardiac troponin I 99th percentile upper reference limits in a healthy Pakistani population

**DOI:** 10.12669/pjms.36.6.2328

**Published:** 2020

**Authors:** Kulsoom Bahadur, Aamir Ijaz, Momin Salahuddin, Aftab Alam

**Affiliations:** 1 Kulsoom Bahadur, MBBS, FCPS. Department of Chemical Pathology and Endocrinology, Resident of Chemical Pathology, Rehman Medical Institute, Peshawar, Pakistan; 2 Aamir Ijaz, MBBS, FCPS. Department of Chemical Pathology and Endocrinology, Professor and Consultant Chemical Pathology, Rehman Medical Institute, Peshawar, Pakistan; 3 Momin Salahuddin, MBBS, FCPS. Professor of Cardiology Department, Rehman Medical Institute, Peshawar, Pakistan; 4 Aftab Alam, MBBS, FCPS. Assistant Professor of Cardiology Department, Rehman Medical Institute, Peshawar, Pakistan

**Keywords:** High sensitive cardiac Troponin I, 99^th^ percentile, Troponin T, Myocardial infarction

## Abstract

**Objective:**

This study aims to establish the 99^th^ percentile upper reference limits of high sensitive cardiac troponin I in a healthy Pakistani population.

**Methods:**

It was an Observational cohort study carried out in Department of Chemical Pathology and Endocrinology Rehman Medical Institute Peshawar, over the period of one year (January 2019- December 2019). Total 299 cardio-healthy males and females were interviewed and taken past medical history. Based on history, clinical examination, echocardiogram and laboratory data including results of estimated glomerular filtration rate (eGFR) and N-terminal pro-B-type natriureteric peptide (NT-proBNP), subjects with possible subclinical diseases were excluded. High Sensitive Cardiac Troponin I (hs-cTtrop I) was analysed on Abbot ARCHITECT STAT ci8200 using chemiluminescent immunoassay technique. The 99th percentile upper reference limit (URL) of hs-cTtrop I was determined using a non-parametric statistic, while gender specific results were compared.

**Results:**

In this study, 178 males (59.5%) and 121 females (40.5%) were included. The median Interquartile ranges (IQR) of age was 57 (11.6) for males and 56 (13) for females. The 99th percentile URL hs-cTtrop I was found to be 33.9 ng/L, while gender specific values were 38.41ng/L and 15.73ng/L for males and females, respectively (p= 0.0045).

**Conclusion:**

High sensitivity cardiac troponin I 99th percentile URL in our study population was found to be 33.9 ng/L with gender specific values being 38.41 ng/L and 15.73ng/L for males and females respectively. Troponin I in males was substantially high in comparison with females.

## INTRODUCTION

Globally Chest pain is among the most common causes for hospitalization. Despite most patients with no evidence of myocardial infarction, hospital admission for observation and serial cardiac troponin testing is required to identify those either with or without myocardial infarction. Early rule-in / rule-out of myocardial infarction (MI) is critical for patient care and resource allocation in patients presenting at the emergency department. The biomarkers of choice for the diagnosis of Acute myocardial injury are cardiac troponin I (cTnI) and cardiac troponin T (cTnT), since they are the most sensitive and cardiac-specific laboratory tests of myocardial injury globally available. High sensitivity cTn (hs-cTn) assays have currently been used in clinical setting.^1^ Introducing high sensitive cTn assays has contributed to improved awareness of myocardial damage other than acute coronary syndromes.^2^ Myocardial injury is by far the most frequent cause of irregular troponin findings, and its incidence is expected to rise with an aging population, increased cardiac comorbidity, and increased troponin assay sensitivity.^3^

Cardiac troponin I (cTnI) and T (cTnT) are regulatory proteins in cardiac tissue that regulate actin and myosin calcium-mediated interactions.^4^ It is a protein that aids in the contraction of cardiomyocytes. Troponin is a heterotrimeric protein consisting of a tropomyosin-binding subunit troponin T (TnT), a Ca^2+^ -binding subunit, troponin C (TnC), and a troponin I (TnI) inhibitory subunit.^5^ The term myocardial injury is used when there is evidence of elevated cardiac troponin values (cTn) with at least one value above the 99th percentile upper reference limit (URL). The myocardial injury is considered acute if there is a rise and/or fall of cTn values.^6^ In the general population, even cardiac troponin values below the URL are correlated with increased mortality and morbidity of the cardiovascular disease (CVD).^7^ To distinguish MI from other cardiac conditions, the rise in troponins is usually constant. cTnT and cTnI tend to be of equal use except in patients with renal failure where cTnT is higher than cTnI. Nearly 100 percent of dialysis patients will have an increased cTnT with high sensitivity assays. In almost the same range as other known cardiovascular risk factors, the last CKD stage (15 mL / min/1.73 m2) is independently correlated with a steeper increase in hs-cTnT over time.^8^

Some of the terms related to analytical performance specifications are defined below to help understand the nature of high sensitivity troponin assay.^9^

### Co-efficient of variation (CV)

is a measure of the assay’s reproducibility (or precision) and is measured as the ratio of the standard deviation over the mean value for repeated testing of the same sample.^9^

### Limit of detection (LoD)

is the lowest observable value as one gradually dilutes a cardiac troponin (cTn) sample. The detection limit has been used as a criterion in a number of studies to rule out myocardial infarction.^9^

### Limit of quantitation (LoQ)

is the concentration at which the CV for the assay is < 20 per cent. The more sensitive and accurate an analysis is, the lower the quantitation limit will be.^9^

### Limit of the blank (LoB)

is the measurement of noise in the system. It is the lowest value that can be distinguished from zero, and is measured as the upper 95 percentile of observed values from repeated blank sample measurements.

### Upper reference limit (URL)

represents the upper limit of a normal population. For cTn, that is known as the normally distributed 99 percentiles, which is about three standard deviations from the mean value. Therefore, 1 percent of normal subjects can have a value above the URL of 99 percentiles.^10^

### High-sensitivity (hs)

The term “high-sensitivity” addresses an assay’s characteristics and not a difference in the forms of cardiac troponin (I or T) being measured. It is important to understand that high sensitivity refers to analytical sensitivity rather than clinical sensitivity in the sense of troponin research. International Federation of Clinical Chemistry (IFCC) has defined two criteria for an assay to be “high-sensitive”. First, the percentage coefficient of variation (%CV) at the 99th percentile URL should be ≤10%. Second, measurable concentrations should be attainable at a concentration at or above the assay’s limit of detection (LoD) for >50% of healthy individuals both men and women.^11^ IFCC Task Force on Clinical Application of Cardiac Biomarkers and AACC Academy have provided a few guidelines for the use of hs troponin assays. Laboratories will test at least three different levels of QC materials per day from below the 99th percentile URL to the upper analytical range. For hs troponin I assay CV% at the 99th percentile URL should be <10%. The lower assay limits (LoB, LoD, and LoQ) must be validated by the clinical labs. Such analytical parameters should be minimally re-validated on an annual basis. Another recommendation is to report hs-cTn in whole numbers, using ng / L without decimal point. One decimal point should be used for reporting the QC values. For contemporary cTn assays, units up to 3 decimal points are recorded in ug/L or ng/ml.^11^

The most important advantage of performing Troponins is its Cardiac Specificity.^12^ Increasing demand for such an early diagnosis of AMI has spurred to many of the cardiac troponin tests with high analytical sensitivity. High-sensitivity, however, means that the assays have much lower LoD for troponin testing, allowing quantification of troponin at levels that were undetectable with previous assays. In addition, by reducing the detection limits for an assay by increasing its analytical sensitivity, there may or may not any effect on the 99th percentile cut-point that defines the upper limit of the normal range.^13^

Hence the current analysis is to determine the 99th percentile upper reference limit for high sensitive cardiac troponin I in a healthy Pakistani men and women.

## METHODS

It was a cohort study carried out in Chemical Pathology Department of Rehman Medical institute Peshawar, from January 2019 till December 2019. Sample size of 299 was calculated using 95% confidence level, 5% margin of error and taking expected frequency of 95^th^ percentile of High Sensitive Cardiac Troponin I i.e. 50% in cardiac patients. The study was approved by Institutional Review Board/ Ethics Review Committee, Rehman Medical institute, Peshawar via letter No. RMI/RMI-REC/Approval 60 on December 21, 2018. Informed consent was obtained.

### Sampling technique

Plasma samples of 100µL, in Lithium heparin tube were taken and analyzed on Abbot Architect using chemiluminescent immunoassay technique, using three levels of controls for Quality control purpose. Non probability consecutive sampling was done. Two hundred ninety-nine apparently cardio healthy individuals of both genders aged 21-90 years were included. Manufacturers claimed analytical performance specifications are LoB: 0.7 - 1.3 ng/L, LoD: 1.1-1.9 ng/L and LoQ: < 10 ng/L with a bias of < 10% and imprecision of < 10%. Demographic details of patients (name, age, sex) were obtained. All people were interviewed and taken past medical history regarding any cardiovascular diseases. Based on history, clinical examination, echocardiogram and laboratory data including results of estimated glomerular filtration rate (eGFR) and N-terminal pro-B-type natriureteric peptide (NT-proBNP), subjects with possible subclinical diseases were excluded.

### Statistical Analysis

Data was analyzed using SPSS version 21.0. None of the assay manufacturers played a role in the design of the research, the interpretation of the data. Quantitative data like age and Troponin I were presented as median and interquartile ranges (IQR). Qualitative data like gender was presented as frequency and percentages. Comparison of hs cTrop I concentration between gender was analyzed using Mann Whitney U test. P-value < 0.05 was considered as significant.

## RESULTS

Among 299 apparently cardio healthy subjects, 178(59.5%) were males and 121(40.5%) were females. The demographics of the data and the median hs-cTtrop I values are sown in Table-I. The median age of male 57(11.6%) and female 56(13.9%). The median age was 56 years old with the oldest being 86 and youngest being 21 years old. Most of the people 246 (82.3%) were above 40 years old. There was no significant difference between age and hs-cTtrop I. Median hscTrop I was 3.9 ng/l and 99^th^ percentile value is 33.9 ng/L. Median hs-cTtrop I in males and females were 4.3 and 3.0 ng/L, while 99^th^ percentile URL were 38.41 ng/L and 15.73 ng/L, respectively. The 99^th^ percentile URL of hs-cTtrop I for the population was 33.9 ng/L. Median hs-cTtrop I concentration were significantly higher in men than in women (p= 0.0045).

**Table-I:** Distribution of Gender, age categories with Troponin I concentration.

		F (%)	Median ng/L (95% CI)	IQR	p-Value
Gender	Male	178 (59.7%)	4.3 (7.31-10.64)	9.83	0.0045
	Female	121 (40.46%)	3.0 (3.92-5.47)	6.15	
Age	< 40	53 (17.7%)	1.7(2.90-6.73)	4.30	0.0055
	≥40	246 (82.2%)	4.3 (3.96-6.43)	7.93	

## DISCUSSION

The concentration of cTn used for MI diagnosis varies greatly in relation to the 99th percentile URL assay by different hospital laboratories. In present-day medicine, the dominance of patient characteristics on biological markers and the implementation to clinical decisions is becoming increasingly important. Quantification of cTn levels is particularly important for an accurate and timely diagnosis of MI. Gender- specific 99^th^ percentile URL variations in cTrop concentration have also been recognized more and more.^14^ Adnan et al suggested the role of cardiac troponin I elevation in the MI diagnosis.^15^ Several studies have shown the manufacture’s 99^th^ percentile URL does not match values seen in large community- based cohort studies and may result in major differences in the determination of the 99^th^ percentile URL. Kimenai et al compared the manufacturers hs-cTnT (Roche) and hs-cTnI (Abbott) threshold with the single sex-specific and age-specific 99th percentile URL reference local cohort. The overall 99th percentile of hs-cTnI and hs-cTnT upper reference limits were 13 and 15 ng / L. The upper reference limits for men were higher than for women (hs-cTnI: 20 vs. 11 ng / L) (hs-cTnT: 16 vs. 12 ng / L) respectively.^16^ The 99th percentile URLs for the Atherosclerosis Risk in Communities (ARIC) study, the Dallas Heart Study (DHS), and the Cardiovascular Health Study (CHS) were 21, 14, and 28 ng / L respectively in population-based studies. The 99th percentile URL value increased with age within each cohort and was higher among men.^17^

**Table-II:** The 99 percentile of Troponin level for overall population according to gender.

	Median (ng/L)	99^th^ percentile (ng/ L)
Total Samples	3.9	33.9 (90% CI 5.99-7.39)
Male	4.3	38.41 (90% CI 7.58-10.54)
Female	3.0	15.73 (90% CI4.05-5.34)

Ninety nine percentile URLs are highly dependent not just on the demographic and physiological variables (i.e. parameters for the apparently healthy reference population), but also on the analytical accuracy of cardiac Troponin methods and on the mathematical algorithms used to measure the 99^th^ percentile URL.^18^ Troponins are ideal markers for early identification of patients with acute coronary syndrome while inflammation markers are useful in diagnosing and evaluating inflammation severity. In patients who were admitted to Emergency Department, who did not present with ACS, detectable troponin I below the 99th percentile is correlated with increased mortality risk at 2-year follow up.^19^ Eggers KM et al has emphasized on the need for a structured statistical method to measure 99th percentile for cardiac trop I and support the use of the non-parametric process and a conservative strategy in outliers identification.^20^ Monitoring of myocardial enzymes and cardiac troponin T plays a significant role in the treatment of children with extreme pneumonia.^21^ In patients with Pompe’s disease, increased serum cTnT levels were observed with skeletal muscle damage, instead of acute myocardial damage. Elevated concentrations of cTnT in Pompe disease as well as other likely neuromuscular disorders should always be considered with due care in order to prevent possible cardiovascular intervention.^22^

Presently implemented clinical decision values for hs-cTtrop I are not biologically equivalent and thus lead to significant disparities in ACS diagnosis. If managed with the alternative hs-cTtrop I assay, one of 5 ACS patients will receive a diagnosis other than AMI.^23^

Differences in the 99th percentile URL values obtained in various studies highlighting the importance of setting a local reference value. Data from Korean population showed, the 99th percentiles of hs-cTtrop I concentrations for all participants were 18 (90% confidence interval [CI], (14–35) ng / L, 19 (90% CI, 11–41) ng / L for females and 20 (90% CI, 15–69) ng / L for males. The median concentrations of hs-cTtrop I in both males and females were significantly higher in participants aged 50 years and older than in those younger than 50.^24^ A strong impact of age on 99^th^ percentile URL has been found by the Gutenberg Health Study.^25^ In a Malaysian study 99^th^ percentile URL was found to be 23.7 ng/L for overall population, and 29.9ng/L and 18.6 ng/L for male and female gender, respectively.^26^ Most probable reason for this discrepancy is that in our study, most of the people were elderly with a median age of 56 years, 82% of the people were above 50 years of age and only 18% of the people were less than 40 years of age.

Our study showed that the 99th percentile URL of hs-cTrop I in an apparently healthy Pakistani population is 33.9 ng/L with variation in gender specific values being 38.41 ng/L and 15.73ng/L for males and females, respectively. The research is confined to the Rehman Medical Institute, where the main bulk of the population is from Peshawar, so the 99th percentile is measured primarily from these individuals and not from the entire population.

## CONCLUSION

High sensitivity cardiac troponin I 99th percentile URL in our study population was found to be 33.9 ng/L with gender specific values being 38.41 ng/L and 15.73ng/L for males and females respectively. Troponin I in males was substantially high in comparison with females.

### Authors’ Contribution:

**KB** conceived, designed and manuscript writing.

**KB, MS and AA** did data collection and statistical analysis.

**AI** did editing, review and final approval of manuscript and he is responsible and accountable for the accuracy or integrity of the work.
